# Metal load and oxidative stress driven by organotin compounds on rainbow trout

**DOI:** 10.1007/s11356-021-12984-w

**Published:** 2021-03-04

**Authors:** Gabriele Magara, Antonia Concetta Elia, Ambrosius Josef Martin Dörr, Maria Cesarina Abete, Paola Brizio, Barbara Caldaroni, Marzia Righetti, Paolo Pastorino, Melissa Scoparo, Marino Prearo

**Affiliations:** 1 Department of Chemistry, Biology and Biotechnology, University of Perugia, via Elce di Sotto 8, 06123 Perugia, Italy; 2 The Veterinary Medical Research Institute for Piemonte, Liguria and Valle d’Aosta, via Bologna 148, 10154 Torino, Italy

**Keywords:** Bioaccumulation, Fish, Liver, Oxidative stress biomarkers, Stress response, Tributyltin

## Abstract

Tributyltin-based (TBT) antifouling paints, widely used for the treatment of flooded surfaces, have been banned in 2008 for their high environmental persistence and bioaccumulation in aquatic organisms. Although it is still present in aquatic ecosystems, oxidative stress driven by TBT has been still poorly investigated in fish. The aim of the study was to examine the time-course stress responses in liver of rainbow trout that received a single intraperitoneal injection of tributyltin chloride (TBTC) or tributyltin ethoxide (TBTE), both at a dose of 0.05 and 0.5 mg/kg. Levels of metallothioneins, total glutathione, malondialdehyde, superoxide dismutase, catalase, glutathione peroxidase and glutathione S-transferase were evaluated at 3 and 6 days post-injection. Tin load was measured in the muscle of the same fish. Differences were observed in the time-course accumulation of tin with a clear dose-response relationship. Although individual oxidative stress biomarkers varied, the biomarker profile indicated different stress mechanisms caused by both TBTC and TBTE. The weak induction of metal-trapping metallothioneins and the changes of oxidative stress biomarkers suggested a stress-pressure in both TBT-treated trout, advising for an ecotoxicological risk for freshwater ecosystems.

## Introduction

Organotin compounds and mainly tributyltin (TBT) are organometals widely employed during the last decades in antifouling paints, due to their broad-spectrum biocide activity (Antizar-Ladislao 2008). Despite being appreciated for their high effectiveness and performance on the hull of boats, their excessive use along with high persistence caused an alarming increase of the concentration of TBTs in harbours and marine waters around the world, from 0 to 2929 ng Sn L^–1^ in Europe (Ritsema et al. 1991), 500 ng Sn L^–1^ in South America (Santos et al. 2009) and 378 ng Sn L^–1^ in North America (Valkirs et al. 1986). Furthermore, TBT has been declared one of the most toxic chemicals ever introduced into the aquatic environment (Goldberg 1986; Zuo et al. 2012; Revathi et al. 2013), leading to devastating biological effects (e.g. immunotoxicity, embryotoxicity, genotoxicity and endocrine disrupting properties) on mussels, fish, marine mammals and birds (Tolosa et al. 1996; Lagadic et al. 2007; Antizar-Ladislao 2008; Lopes-dos-Santos et al. 2014; Filipkowska and Lubecki 2016). Consequently, the use of TBT in antifouling coats was limited by the European Community since 2003, and it was banned by the International Maritime Organization (IMO) since 2008 (https://www.imo.org/). Nevertheless, after having banned, high environmental concentrations of TBT are still found in marine and freshwater ecosystems (Gao et al. 2017; Okoro et al. 2016; Quintas et al. 2019). In Argentina and South Africa, TBT concentrations in seawater ranged from 21.4 to 387 ng/L (Quintas et al. 2019) or 67 to 111, 290 ng/L, respectively (Okoro et al. 2016). Furthermore, a recent study reporting for the first time the spatiotemporal variability of TBT in surface freshwater under dynamic water level conditions in the Three Gorges Reservoir Region in China showed an extensive spatial distribution of TBT compounds across the investigated areas, and a maximum of 393.35 ng Sn/L in Zigui, the downstream region (Gao et al. 2017). These studies indicate an enduring release of TBT into marine and freshwater ecosystems and can be yet persisting in aquatic environments and in the food chain especially in those areas in which their usage has not been totally restricted (Gao et al. 2020). Therefore, the ecological risk driven by TBT can still represent a current issue for aquatic populations and ecosystems, particularly in freshwater, and the related scientific field of research.

The impact of TBTs compounds has been widely and strongly investigated in marine environments; however, less consideration was expressed for the environmental risk of these chemicals in freshwater ecosystems (Martínez et al. 2017). Although several toxicity mechanisms following TBT exposure have been widely elucidated in fish species, such as disruption of steroidogenesis in zebrafish *Danio rerio* (McGinnis and Crivello 2011) and brown trout *Salmo trutta* (a Marca Pereira et al. 2014) or reproductive effects such as infertility in Japanese whiting *Sillago japonica* (Shimasaki et al. 2006) and inhibition of gonad development in rockfish *Sebastiscus marmoratus* (Zhang et al. 2009), others are still debated. In this context, oxidative stress driven by TBT is still poorly investigated in fish (Zhang et al. 2017a, b). As previously reported, TBT can lead to apoptosis activating the caspase-dependent pathway via inositol 1,4,5-triphosphate and ryanodine, following an overproduction of reactive oxygen species (ROS) (Nakatsu et al. 2007). The latter can also lead to severe oxidative damage (Nakatsu et al. 2007), such as lipid peroxidation, protein oxidation and DNA damage (Bernat and Długoński 2012; Ishihara et al. 2012). Increased doses of tributyltin chloride (TBTC) were correlated with a higher malondialdehyde (MDA) concentration, a toxic by-product of lipid peroxidation and a decreased activity of the main antioxidant enzymes, lysozyme and the content of immunoglobulin M in the liver of *D. rerio* (Zhang et al. 2017a). Changes of lipid accumulation, oxidative stress and immune-toxic effects were also confirmed by Zhang et al. (2017b) in the muscle of rare minnow *Gobiocypris rarus*, concluding that TBT may affect growth and health status of fish. Despite these studies, to our best knowledge, there are no studies on the effects of different tributyl compounds on the levels of oxidative stress biomarkers in rainbow trout *Oncorhynchus mykiss.*

In aquatic species, the levels of those biomolecules involved in antioxidant and detoxifying defence can be used as informative biomarkers to investigate the oxidative stress exerted by xenobiotics (Al Kaddissi et al. 2012, 2014; Cozzari et al. 2015; Elia et al. 2007, 2017, 2018, 2019, 2020; Magara et al. 2018, 2019). Metallothioneins (MTs) are proteins able to bind heavy metals, acting as homeostatic buffers. Superoxide dismutase (SOD), catalase (CAT), glutathione peroxidase (GPx) and reduced glutathione (GSH) are involved in the defence against the oxidative damages to biological macromolecules transforming ROS in non-reactive compounds. However, when antioxidant shield fails, free radicals can lead to oxidation of biological membranes, following loss of cell stability and overproduction of MDA. Glutathione S-transferase (GST) is a phase II enzyme that catalyses the conjugation of GSH to the electrophilic centres of a wide range of substrates by sulfhydryl groups, preventing their interaction with biomolecules.

In this study, the rainbow trout *O. mykiss* was used as experimental model recognised as standard, highly sensitive species used in both regulatory testing and risk assessment (Teather and Parrott 2006). The aim was to elucidate the time-course pro-oxidant effects of two tributyltin compounds (tributyltin chloride, TBTC, and tributyltin ethoxide, TBTE) in the liver of this fish species. TBTC and TBTE were chosen to investigate the possible different antioxidant mechanisms related to the different compounds. A biomarker approach was carried out, measuring the levels of MTs, total glutathione, MDA, SOD, CAT, GPx and GST. Tin accumulation was recorded in trout muscle.

## Materials and methods

### Chemical preparation and rational

Tributyltin chloride (code T50202, purity 96%) and ethoxide (code 251518, purity 97%) were purchased from Sigma-Aldrich (Italy). Chemicals were administered by intraperitoneal injection, in order to minimise the intra-species uptake variability. Experimental concentrations of tributyltin were set considering two theoretical concentrations of 100 and 1000 ng/l, which have been reported as sufficient to cause adverse effects on the reproductive cycle of aquatic organisms (Horiguchi et al. 2002; Revathi et al. 2014a, b). Then, we based on the bioconcentration factor (BCF) in rainbow trout of 406 for TBT (64 days, Martin et al. 1989) to derive the expected dose of compounds inside fish after a prolonged exposure. This experimental approach allows reproducing the biochemical-molecular stress responses of a long-term exposure in a shorter experimental period.

### Tributyltin treatment

A single lot of rainbow trout was purchased from a local fish farm. Ninety-six females (mean ± SD body weight 350.2 ± 10.9 g; total length 31.9 ± 2.8 cm) were acclimated for 10 days in eight flow-through 1500-L indoor fiberglass tanks (12 specimens each) filled with artesian well water (mean water temperature 13.5°C, dissolved oxygen 8.7 mg/l) at the Experimental Station of the Department of Agricultural, Forest, and Food Sciences (DISAFA), University of Turin (Italy).

Prior the experiment, a preliminary 1-week treatment was carried out to set the experimental dosages of TBT. A stock solution of 5 mg/ml was prepared for each TBTC and TBTE compounds dissolved in DMSO. Twenty-four fish were anaesthetised with ethyl 3-aminobenzoate methane sulphonate (MS 222; 100 mg/L, Sigma-Aldrich, St. Louis, MO) and then given an i.p. injection of an appropriate volume of stock solution to obtain an internal dose of 0.5 mg/kg TBT. As tributyltin compounds were dissolved in DMSO, each control fish received an i.p. injection of the carrier. All rainbow trout survived during the 1-week trial.

Therefore, we selected two TBT dosages for the treatment conditions: 0.05 and 0.5 mg/kg TBTC or TBTE. At the start of the experiment (T0), sixty rainbow trout were anaesthetised with MS-222 (100 mg/L), and then given a single i.p. injection of TBT at the chosen doses. The five experimental groups (12 fish, respectively) were tributyltin chloride (TBTC, 0.05 and 0.5 mg/kg), tributyltin ethoxide (TBTE, 0.05 and 0.5 mg/kg) and one control group. The control group received only the DMSO. The treatment was conducted under the same acclimatisation conditions. Specimens were fed twice a day with commercial diet (Skretting) at 1% of body weight (bw)/day. No lethal effects were recorded. At 3 and 6 days post injection, six fish from each treated and control group were collected and euthanised with an overdose of MS-222 (250 mg/L). Each fish was weighed and total length measured. Liver and muscle were removed, washed in saline solution, dried and stored immediately at −80°C for biochemical and chemical analysis.

### Chemical analyses

Dorsal fillet samples were collected from each specimen. For digestion, 1.5 g of each tissue was placed into a Teflon pressure vessel, and concentrated nitric acid and hydrogen peroxide 30% v/v were added. The samples underwent microwave digestion for mineralisation. After cooling, the digested samples were transferred into 50-ml measuring tubes. The inner walls of the Teflon pressure vessels were rinsed with ultrapure distilled water, and the rinse water was transferred into the tubes. Ultrapure distilled water was then added to the tubes to a final volume of 50 ml. Quantification of copper and tin was performed by a Thermo Xseries II ICP-MS instrument (Thermo Scientific, Germany) equipped with a CETAC ASX 500 Model 520 (CETAC Technologies, USA) auto sampler and a peristaltic pump nebuliser. Accuracy of analysis was tested using certified reference material (NRCC-DORM-2 Dogfish muscle, National Research Council of Canada, Ottawa, Canada). Concentrations measured in reference material fulfil the range of the certified values. LOQ of method was 0.020 mg/kg for both copper and tin.

### Biochemical analyses

All assay conditions for oxidative stress biomarkers were performed according to the original methods as previously reported in Elia et al. (2017).

*Metallothionein* (MT) levels were measured in 3 pooled samples of two livers each for a total of 6 individuals per treatment group. Samples were homogenised (1:4) in a buffer containing 0.02 M 2-amino-2-hydroxymethylpropane-1,3-diol (TRIS/HCl), 0.5 M sucrose, 0.1 mg/ml bacitracin, 0.008 TIU/ml aprotinin, 87 μg/ml phenylmethylsulfonyl fluoride (PMSF) and 0.1 μl/ml α-mercaptoethanol. The homogenates were centrifuged at 14500×*g*, and the cytosolic fraction was added with chloroform/ethanol and HCl/ethanol to obtain the partially purified MTs fraction. Pellets were washed with ethanol/chloroform/Tris–HCl solution (87:1:12) and suspended in 0.25 M NaCl. A destabilizing solution (HCl 1N + ethylenediaminetetraacetic acid [EDTA] 4 mM) and Ellman’s reagent (DTNB, 5,5′-dithiobis-(2-nitrobenzoic acid)) were added to each sample. The absorbance was measured at 412 nm and compared with that obtained from a standard curve with reduced glutathione (1 mg/ml GSH). MTs are reported as μg/g of tissue.

*Malondialdehyde* (MDA) levels were analysed in liver according to the method previously reported in Pacini et al. (2013). Each liver sample (0.2 g) was homogenised (1:10) in 20 mM 2-amino-2-hydroxymethylpropane-1,3-diol (Tris/HCl), pH 7.4 and 0.5 M hydroxytoluenebutylate (BTH), and then centrifuged at 3000×*g* for 20 min at 4°C. The supernatant was derivatized in 1-methyl-2-phenylindole (10.32 mM in acetonitrile/methanol 3:1), HCl and buffer solution (Tris/HCl, pH 7.4) with standard sample or MDA (0–4 μM of 1,1,3,3-tetramethoxypropane). All samples were incubated at 45°C for 60 min, then centrifuged at 15,000×*g* for 10 min and read spectrophotometrically at 586 nm. MDA is reported as nmol/g of tissue.

*Total glutathione* (GSH+2GSSG) was measured in each sample according to the method reported in Dörr et al. (2020). Liver was homogenised (1:5) in 5% sulfosalicylic acid with 4 mM EDTA, then centrifuged at 30,000×*g* for 15 min. The assay was performed in potassium phosphate (KP) buffer 100 mM pH 7, EDTA 1 mM, NADPH 4 mg/ml and DTNB 1.5 mg/ml both dissolved in 0.5% NaHCO_3_ and GR 1U. The oxidised glutathione was used as the standard, and the absorbance was read at 412 nm. GSH+2GSSG is reported as nmol/g of tissue.

For enzyme analyses, liver (0.2–0.3 g) of each trout was homogenised (1:10 w/v) in 100 mM Tris buffer, pH 7.8, 100 μM phenylmethylsulfonyl fluoride (PMSF), 0.1 mg/ml bacitracin and aprotinin 0.008 TIU/ml. Then, the samples were centrifuged at 18,000×*g* for 20 min and at 50,000×*g* for 1 h at 4°C to obtain the cytosolic fraction. The analysis of SOD was performed in 50 mM Na_2_CO_3_ + EDTA 0.1 mM pH 10, 500 mM cytochrome C, 1 mM hypoxanthine and xanthine oxidase. The reduction of cytochrome C by the xanthine/hypoxanthine system was measured at 550 nm with a standard curve of SOD units. SOD is reported as U/mg of protein. CAT activity was measured at 240 nm using 100 mM sodium phosphate buffer (NaH_2_PO_4_ + Na_2_HPO_4_) pH 7, 12 mM hydrogen peroxide (H_2_O_2_) and sample. CAT is reported as μmol/min/mg of protein. GPx activity was determined at 340 nm using 100 mM NaH_2_PO_4_ + Na_2_HPO_4_ buffer + EDTA 1 mM pH 7.5, 1 mM sodium azide (NaN_3_), 0.12 mM NADPH, 2 mM GSH, 1U GR, 0.6 mM H_2_O_2_ and sample. GPx is reported as nmol/min/mg of protein. GST activity was measured at 340 nm using 100 mM NaH_2_PO_4_ + Na_2_HPO_4_ buffer pH 6.5, 1 mM GSH, 1 mM 1-chloro-2,4 dinitrobenzene (CDNB) and sample. GST is reported as nmol/min/mg of protein. Protein concentration was determined according to the method of Lowry et al. (1951) and used to normalise the cytosolic enzyme levels. All biochemical analyses were performed in triplicate for each liver sample with a Varian spectrophotometer (Cary 50 Thermostat Cell Holder) at a constant temperature of 25°C.

### Statistical analysis

Data are reported as mean and standard deviation (SD). Normality of all data was determined using the Shapiro-Wilk’s test. Statistical analysis of significance was evaluated following two-way ANOVA (*p* <0.05) with Tukey’s multiple comparisons test. Statistical evaluations were performed using GraphPad Prism software. Principal component analysis (PCA) was performed to check for trends in biomarkers levels and Sn concentration considering both control and treated groups (TBTC and TBTE) at 3 and 6 days post injection. The PCA results were plotted using open-source data analysis software RStudio® version 1.1.463 (RStudio, Inc.)

## Results

### Chemical analysis

**Fig. 1:**
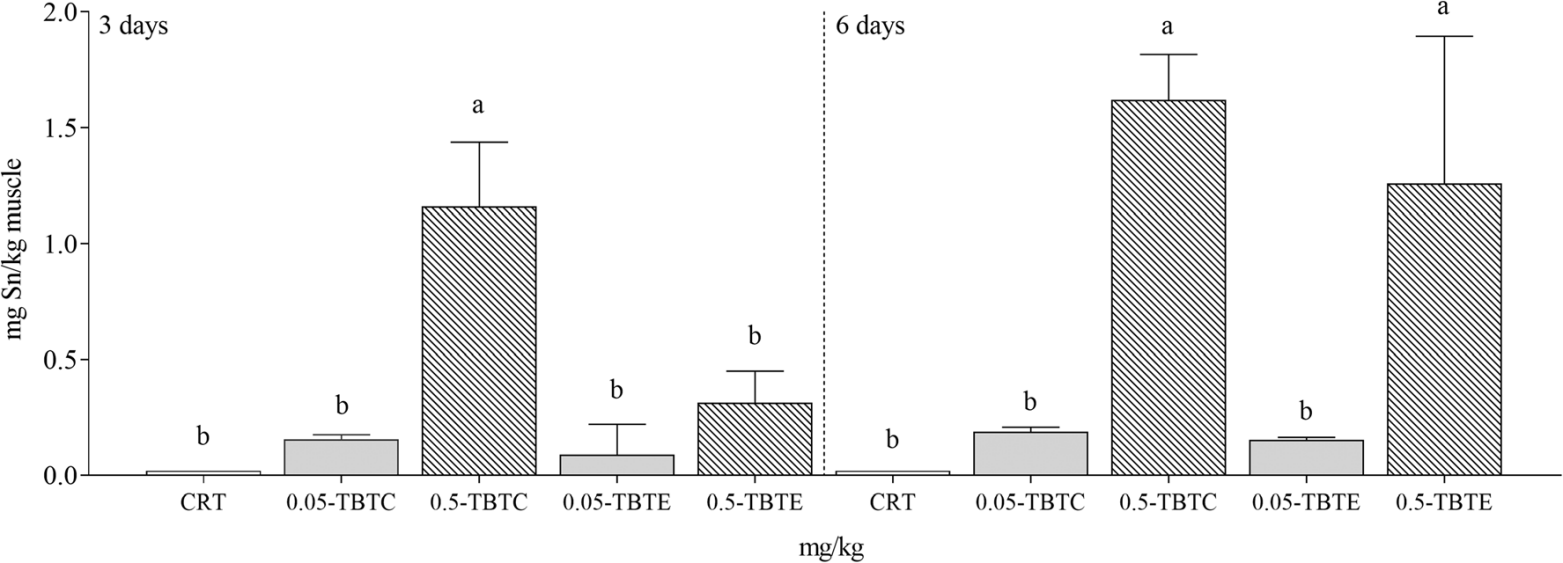
Tin concentration in muscle of rainbow trout treated with tributyltin chloride and tributyltin ethoxide (0.05 and 0.5 mg/kg of each compound). CRT control, TBTC tributyltin chloride, TBTE tributyltin ethoxide. Muscle of each group was analysed individually. Different letters (a, b, c) indicate statistical significant differences (*p*<0.05) between experimental groups (CRT, TBTC and TBTE) at the same time point

During the trial, no lethal effects were recorded. Tin concentration in control muscle did not markedly vary (3 and 6 days <0.020 mg/kg, *n* = 6) at both endpoints. A dose-dependent increase of tin levels (sevenfold) was recorded in muscle of both TBTs with an early metal load measured in TBTC fish (Fig. 1).

### Biochemical analysis

**Fig. 2:**
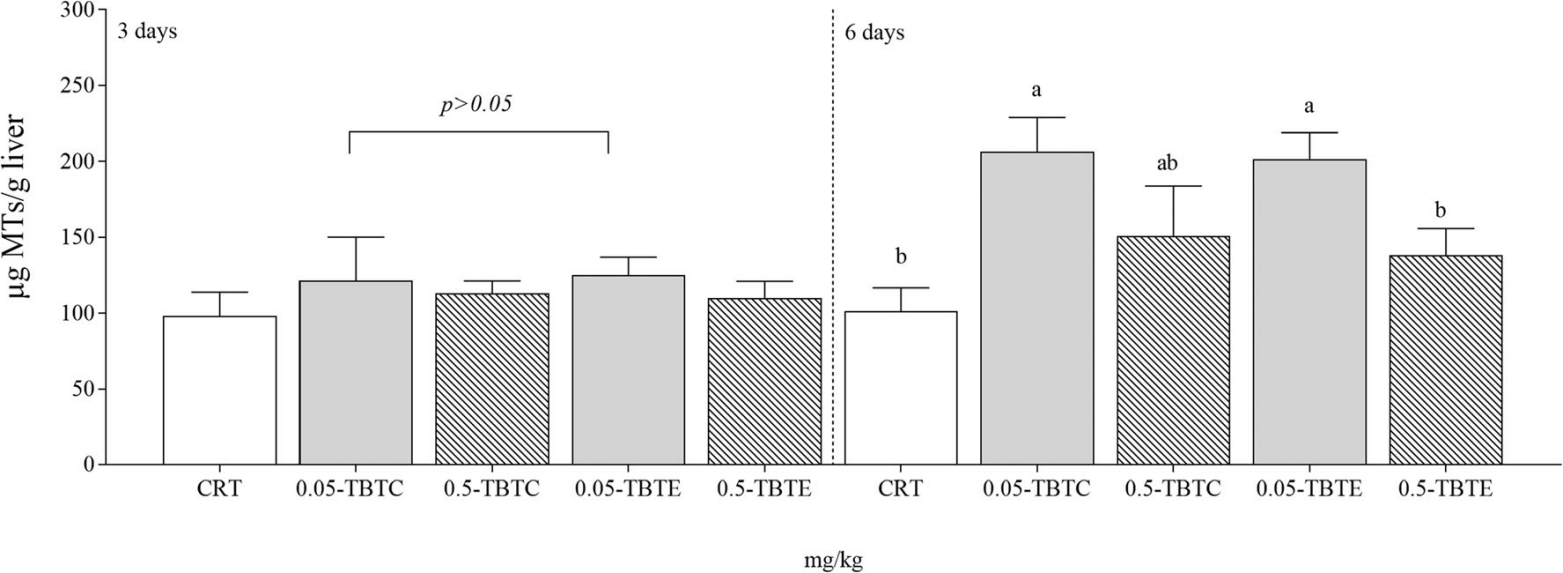
Metallothionein concentration in liver of rainbow trout treated with tributyltin chloride and tributyltin ethoxide (0.05, 0.5 mg/kg of each compound). CRT control, TBTC tributyltin chloride, TBTE tributyltin ethoxide. Liver of each group was analysed individually. Different letters (a, b, c) indicate statistical significant differences (*p*<0.05) between experimental groups (CRT, TBTC and TBTE) at the same time point

Metallothionein levels increased significantly at 6 days in trout treated with the lower dose of both TBT compounds (twofold) (Fig. 2); two-way ANOVA analysis (Table 1) showed significant effect on time (*p* = 0.0017), treatment (*p* = 0.0016) and interaction (*p* = 0.0334). At 3days, MDA levels were higher in trout exposed to the highest concentration (0.5 mg/kg) of TBTC and TBTE (threefold) and at 6days remained still high (onefold) only in TBTE specimens (Table 2). For this biomarker, effect of treatment (*p* < 0.0001) was found significant (Table 1). No changes in total glutathione levels were recorded among treatments and control groups in both experimental endpoint (Table 2), although effect of time (*p* = 0.0015) and interaction (*p* = 0.0253) between time and treatment emerged (Table 1). At 6days, a lower SOD enzyme activity (50%) was measured in fish exposed to 0.5 mg/kg of both tributyltin compounds (Table 2). The results of two-way ANOVA showed significant effect on time (*p* = 0.0004), treatment (*p* = 0.0137) and interaction between time and treatment (*p* = 0.0114) in liver (Table 1). At 3days, CAT levels increased (50–90%) following both TBTC concentrations (0.05 mg/kg and 0.5 mg/kg), and a 60% reduction was observed in TBTE-exposed fish (0.05 mg/kg) (Table 2). Highly significant effect (*p* < 0.0001) of treatment and interaction on enzyme activity was recorded (Table 1). GPx activity increased at 3days (80%) following both TBTC concentrations and at 6days (onefold) only in trout treated with 0.5 mg/kg TBTC (Table 2); a significant effect (*p* = 0.0003) was found only for treatment (Table 1). At 0.5 mg/kg, GST levels increased following TBTC (30%) and was lowered following TBTE treatment (50%) (Table 2). The results of two-way ANOVA showed significant effect on time (*p* = 0.0106), treatment (*p* = 0.0036) and interaction between time and treatment (*p* < 0.0001) in the analysed tissue (Table 1).

**Table 1:** The results of two-way ANOVA of time, treatment and interaction (time × treatment) of tributyltin chloride (TBTC) and tributyltin ethoxide (TBTE) on oxidative stress biomarkers in liver of rainbow trout

	*F* time	Time	*F* treatment	Treatment	*F* interaction	Interaction
MTs	1, 2	585.10**	4, 8	12.62**	4, 8	4.52*
MDA	1, 3	0.32	4, 12	43.83***	4, 12	2.16
GSH+2GSSG	1, 5	39.87**	4, 20	2.09	4, 20	3.50*
SOD	1, 5	68.10***	4, 20	4.11*	4, 20	4.30*
CAT	1, 5	0.29	4, 20	33.54***	4, 20	12.78***
GPx	1, 5	4.14	4, 20	8.71***	4, 20	2.75
GST	1, 5	15.77*	4, 20	5.55**	4, 20	15.60***

*F* indicates the *F* values following two-way ANOVA test

*MTs* metallothioneins, *MDA* malondialdehyde, *GSH+2GSSG* total glutathione, *SOD* superoxide dismutase, *CAT* catalase, *GPx* glutathione peroxidase, *GST* glutathione S-transferase

Significant code ***p <0.001; ** <0.01; * <0.05

**Table 2:** Oxidative stress biomarkers in liver of rainbow trout treated with tributyltin chloride (TBTC) and tributyltin ethoxide (TBTE)

	Days	CRT	TBTC 0.05 mg/kg	TBTC 0.5 mg/kg	TBTE 0.05 mg/kg	TBTE 0.5 mg/kg
MDA	3	16.41 ± 6.88c	22.69 ± 5.01bc	28.68 ± 6.33b	27.39 ± 8.40b	47.03 ± 8.56a
	6	20.90 ± 8.76b	27.89 ± 8.04b	24.78 ± 2.13b	26.57 ± 3.29b	41.07 ± 7.94a
GSH+2GSSG	3	1339.78 ± 376.54a	1652.02 ± 397.69a	1663.85 ± 227.50a	1429.44 ± 227.24a	1223.65 ± 268.53a
	6	1303.85 ± 235.29a	1236.43 ± 204.65a	878.74 ± 97.94a	1248.91 ± 101.26a	1160.61 ± 162.17a
SOD	3	15.05 ± 1.99a	16.50 ± 3.13a	15.24 ± 3.13a	12.91 ± 1.47a	15.61 ± 1.74a
	6	12.70 ± 2.94a	11.58 ± 3.13ac	7.05 ± 1.70b	10.28 ± 1.49ab	7.59 ± 1.55bc
CAT	3	156.03 ± 26.78c	219.29 ± 18.31a	259.16 ± 50.04a	83.01 ± 10.83b	112.56 ± 28.15bc
	6	170.13 ± 13.66a	175.10 ± 26.14a	167.22 ± 33.44a	143.67 ± 28.86a	160.31 ± 10.31a
GPx	3	25.28 ± 4.33b	39.75 ± 2.80a	40.04 ± 9.82a	26.13 ± 3.50b	33.54 ± 8.44ab
	6	29.47 ± 8.23b	35.30 ± 9.10b	51.67 ± 6.52a	34.78 ± 8.65b	32.12 ± 8.69b
GST	3	136.41 ± 11.98c	123.32 ± 8.36bc	188.62 ± 18.00a	107.59 ± 20.34bc	87.07 ± 5.95b
	6	153.50 ± 23.04a	139.06 ± 17.30a	124.17 ± 27.06a	146.95 ± 27.08a	155.59 ± 24.91a

Liver of each group was analysed individually. Data are reported as mean and standard deviation. Different letters (a, b, c) indicate statistically significant differences (*p*<0.05) between control and treated groups (TBTC or TBTE) at the same time point

*MDA* malondialdehyde, *GSH+2GSSG* total glutathione, *SOD* superoxide dismutase, *CAT* catalase, *GPx* glutathione peroxidase, *GST* glutathione S-transferase, *CRT* control

### PCA analysis

**Fig. 3:**
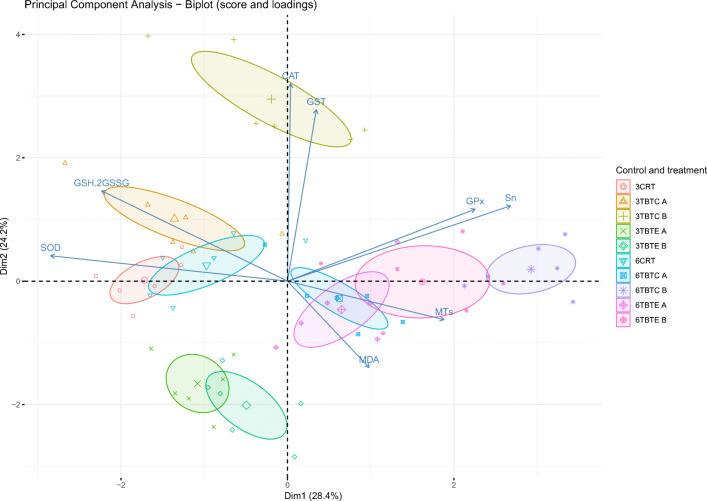
Biplot of score and loadings from principal component analysis. The scores of each group (CRT control, TBTC tributyltin chloride, TBTE tributyltin ethoxide) are denoted by a symbol (largest symbol = average value); 3 = 3-day post injection; 6 = 6-day post injection; A = 0.05 mg/kg; B = 0.5 mg/kg. Confidence ellipses (95%) plot values of each group

The PCA analysis (Fig. 3) showed that the first (Dim1) and the second (Dim2) components accounted for meaningful amounts of the total variance (52.6%): PC1 explained 28.4 % of the total variance and was positively correlated to GPx and Sn, and negatively correlated to GSH+2GSSG and SOD. This outcome suggests that these variables contrast with each other. If we have an increase (or a decrease) of one, then the other one tends to increase (or decrease). PC1 can be defined as a measure of time of exposure, since all 6-day post injection groups are located in the right part of the plot in relation to increasing concentration of Sn and GPx activity. On the other hand, SOD activity is located in the left part, since it decreased at 6 days post injection. PC2 explained 24.2% of the total variance and was positively correlated with GST and CAT. PC2 can be defined as a measure of the treatment. The control (CRT) and treatment (TBTE and TBTC) groups are arranged according to biomarkers levels. Control groups (3 and 6 days post injection) and liver samples at 3 days post injection of 0.05 mg/kg TBTC are located in the left part of the plot, in correspondence to higher levels of SOD and GSH+2GSSG. Instead, liver samples from fish at 6 days post injection of both TBTE and TBTC concentrations (0.05 mg/kg and 0.5 mg/kg) are in the right part of the plot, in relation to higher values of MTs, GPx and Sn. Liver samples at 3 days post injection of both TBTE concentrations (0.05 mg/kg and 0.5 mg/kg) are clearly separated from the other, since they are located on the lower left part of the plot, indicating lower correlation with all biomarker levels. On this path, also samples at 3 days post injection of 0.5 mg/kg TBTC are located in the central higher part of the plot, in relation to higher levels of CAT and GST.

## Discussion

In the present study, the effects induced by both tributyltin compounds (TBTC and TBTE) in rainbow trout were assessed after administration of a single i.p. injection. The contamination route used in our study did not simulate the realistic metal exposure in wild fish; however, it may be considered a useful approach to reduce confounding factors such as inter-individual variability of metal uptake and related biological responses. We measured the chemical and biochemical changes for 6 days, assuming that after one antifouling compound injection, fish would show an early response.

Time/dose-dependent accumulation of tin in muscle was recorded in TBT-treated trout. This outcome is in line with previous studies on marine and freshwater fish (Harino et al. 2000; Martin et al. 1989; Oshima et al. 1998) and was expected, since it is well known that tributyltin bioconcentration factor (BCFs) in fish is high, ranging from hundreds to several ten thousand (Harino et al. 2000). In particular, Martin et al. (1989) showed that in rainbow trout, *Salmo gairdneri* TBT can accumulate in tissues with a BCF of 406. Whether it is assimilated through gills, food or by injection, TBT accumulation in fish may vary (Martin et al. 1989; Oshima et al. 1998). As reported by Martin et al. (1989), TBT accumulation in rainbow trout was in the order of liver > gill > blood > muscle after 15 days of exposure. On the contrary, Oshima et al. (1998) showed a significantly higher concentration of TBT in serum than for liver and muscle in Japanese flounder *Paralichthys olivaceus* and dab *Limanda yokohamae*, when administered intraperitoneally with TBT. However, in both studies, accumulation in muscle resulted lower than for liver or blood. This evidence, along with the high dose-dependent accumulation we measured in muscle of rainbow trout, suggests a very high concentration of TBT in fish tissues and related biological adverse effects in trout chronically exposed to environmental concentrations between 100 and 1000 ng/L (corresponding to 0.05 and 0.5 mg/kg), which are compatible with most of the concentrations of TBT found in the Three Gorges Reservoir region in China, with a maximum of 393.35 ng Sn/L in Zigui (Gao et al. 2017). Moreover, the bioaccumulated TBT may be transferred via reproduction to offspring (Nakayama et al. 2005). In our case, high tin muscle level in TBTs may also suggest an alarming scenario of rising of TBTs along the food-web and even spread in terrestrial ecosystems. In fact, rainbow trout is predated by several carnivorous species, including birds such as white pelican *Pelecanus erythorhynchos* and double-crested cormorant *Phalacrocorax auritus* (Meyer et al. 2016). Therefore, it is feasible that the spread of TBT load to terrestrial ecosystems may lead to several harmful effects related to these compounds even in non-aquatic species, such as oxidative damages, endocrine disruption and adipogenesis effect (Lyssimachou et al. 2017).

Oxidative stress is the final outcome of a multistep process occurring when pro-oxidant and antioxidant mechanisms are imbalanced. In this study, we elucidated for the first time the different effects of TBTC and TBTE on levels of oxidative stress biomarkers in rainbow trout *O. mykiss*. PCA analysis provided a combination of the score (liver samples) and loading plots (biomarkers), contributing to comprehend differences between treatment groups. In particular, it was highlighted the separation between the treatment groups in relation to the time of exposure which in turn affected the biomarkers levels investigated. The levels of hepatic biomarkers were also altered following TBT concentrations. Furthermore, each tributyltin compound differently modulated the biochemical responses, suggesting a compound-dependent regulation of antioxidant mechanisms.

MTs are documented as metal-chelating molecules preventing the imbalance of antioxidant systems and consequently lipid peroxidation (Cirillo et al. 2012). Therefore, the delayed increase of MTs levels in the present study and the subsequent weak activation of this key defence line against metal toxicity resulted rather unexpected. At our best knowledge, information on the mechanisms inducing MTs following TBT exposure in rainbow trout is lacking. Already assessed is that genes coding MTs are differently expressed in response to the type of metal (Jahroudi et al. 1990). Therefore, the diverse MTs response to each metal is not an unusual outcome. In this study, although we did not measure tin load in liver, high metal level at 6 days in muscle leads to assume that the metal has not been adequately entrapped by MTs, following oxidative stress.

MDA is universally considered a biomarker of oxidative damage in fish, and previous studies showed a positive correlation between biomarker levels and TBT exposure in fish and rats (Wang et al. 2006; Mitra et al. 2014; Zhang et al. 2017a, b). Similarly, in our study, TBTC and TBTE affected lipid peroxidation showing early increased MDA levels in trout treated with the higher concentrations. Although the exact mechanism of TBT-induced dysfunction remains to be elucidated, lipid peroxidation could be related to the ability of tributyltin compounds to stimulate ROS production through the activation of the mechanism of apoptosis caspase-dependent, mediated by Ca^2+^ (Nakatsu et al. 2007). Moreover, the increased MDA content in rainbow trout may also be related to dibutyltin (DBT), as a degradation product of TBT. Indeed, a mechanistic study reported the ability of DBT to induce oxidative stress increasing intracellular ROS, mitochondrial mass and mitochondrial ROS (Abouelregal 2014).

It is well known that lipid peroxidation products in fish may be linked to upregulations of several antioxidant enzymes such as SOD, CAT and GPx (Bagnyukova et al. 2006). Mechanistic study on rat reported that TBT can directly inhibit the expression of the prime molecular target against oxidative stress Nrf2-antioxidant system (Mitra et al. 2013). However, previous studies on fish showed different effects of TBT on antioxidant enzyme functionality, related to different doses or animal species (Zhang et al. 2017a, b). Indeed, reduction of SOD, CAT and GPx levels has been described in zebrafish liver exposed to 10–100 ng/L TBTC (Zhang et al. 2017a) whereas in the muscle of rare minnow exposed to 1–10 ng/L TBT, enzyme activity was found higher than in control group (Zhang et al. 2017b). Our results on rainbow trout may suggest that both organotin molecules prompted an overload of the two harmful species such as superoxide anion and hydrogen peroxide. Early enhancement of CAT and GPx activity in TBTC-trout may account for a boosted antioxidant defence, although both enzymes offset oxidative pressure only at lower TBTC concentration, as also suggested by MDA contents. On the other hand, an antioxidant compensation mechanism was observed in fish treated with TBTE. Indeed, previous studies on aquatic organisms have shown that, when CAT is inhibited, GPx can also fulfil its role (Al Kaddissi et al. 2014; Cozzari et al. 2015). However, the higher MDA levels measured in TBTE-treated trout at 6days, as well as the higher muscle tin concentration, suggested a severe biological disorder caused by this organotin compound.

Effects of TBT on GST activity were studied during the last decades on fish, showing contrasting results (Al-Ghais and Ali 1999; Padrós et al. 2003; Wang et al. 2005, 2006; Wu et al. 2007). In liver of *Secastiscus marmoratus*, GST activity was induced after exposure to 19.3 μg/kg TBT for 4 days (Wang et al. 2005), whereas the opposite outcome was observed in arctic charr (*Salvelinus alpinus*) exposed to 0.3 mg/kg TBT (Padrós et al. 2003). On the other hand, Wang et al. (2006) found different enzyme trend following different TBTC doses, with an induced GST activity in liver of *S. marmoratus* exposed to 0.5 and 1 mg/kg TBTC and decreased one in fish treated with 5 and 10 mg/kg TBTC. These previous results suggest a different response of GST to TBT exposure, which may be related to different doses or fish species. In the present study, GST levels were oppositely altered at 3days in specimens treated with the highest concentrations of both tributyltin compounds, increasing in TBTC-treated fish and decreasing in TBTE group. Unlike TBTC, TBTE provided an early, transient but severe effect on GST in rainbow trout, and then a return to the control enzyme level at 6 days indicating a restoration of the detoxifying defence. Both TBT compounds exert a time-related effect on GST activity. Moreover, our results not only confirm the presence of a threshold concentration of TBT to inhibit GST activity in fish, but also suggest a compound-dependent relationship.

## Conclusions

The present findings provide the evidence that both tributyltin compounds may alter the levels of oxidative stress biomarkers in a different manner, indicating that biological responses can be related to dose, time and TBT compounds. Moreover, hepatic MTs levels, as well as the concentration of tin detected in muscle, suggested a failure of this pivotal biological defence against metal toxicity. Further laboratory and field studies are needed to have a deep knowledge about tributyltin dynamics in different aquatic populations and ecosystems.

## Data Availability

The datasets generated during and/or analysed during the current study are available from the corresponding author on reasonable request.

## References

[CR1] a Marca Pereira ML, Eppler E, Thorpe KL, Wheeler JR, Burkhardt-Holm P (2014). Molecular and cellular effects of chemicals disrupting steroidogenesis during early ovarian development of brown trout (*Salmo trutta fario*). Environ Toxicol.

[CR2] Abouelregal AMZAE (2014). Dibutyltin promotes oxidative stress and increases inflammatory mediators in BV-2 microglia cells. Toxicol Lett.

[CR3] Al Kaddissi S, Legeay A, Elia AC, Gonzalez P, Camilleri V, Gilbin R, Simon O (2012). Effects of uranium on crayfish *Procambarus clarkii* mitochondria and antioxidants responses after chronic exposure: what have we learned?. Ecotoxicol Environ Saf.

[CR4] Al Kaddissi S, Legeay A, Elia AC, Gonzalez P, Floriani M, Cavalieri I, Massabuau JC, Gilbin R, Simon O (2014). Mitochondrial gene expression, antioxidant responses, and histopathology after cadmium exposure. Environ Toxicol.

[CR5] Al-Ghais SM, Ali B (1999). Inhibition of glutathione S-transferase catalyzed xenobiotic detoxication by organotin compounds in tropical marine fish tissues. Bull Environ Contam Toxicol.

[CR6] Antizar-Ladislao B (2008). Environmental levels, toxicity and human exposure to tributyltin (TBT)-contaminated marine environment. A review. Environ Int.

[CR7] Bagnyukova TV, Chahrak OI, Lushchak VI (2006). Coordinated response of goldfish antioxidant defenses to environmental stress. Aquat Toxicol.

[CR8] Bernat P, Długoński J (2012). Comparative study of fatty acids composition during cortexolone hydroxylation and tributyltin chloride (TBT) degradation in the filamentous fungus *Cunninghamella elegans*. Int Biodeterior Biodegrad.

[CR9] Cirillo T, Cocchieri RA, Fasano E, Lucisano A, Tafuri S, Ferrante MC, Carpenè E, Andreani G, Isani G (2012). Cadmium accumulation and antioxidant responses in *Sparus aurata* exposed to waterborne cadmium. Arch Environ Contam Toxicol.

[CR10] Cozzari M, Elia AC, Pacini N, Smith BD, Boyle D, Rainbow PS, Khan FR (2015). Bioaccumulation and oxidative stress responses measured in the estuarine ragworm (*Nereis diversicolor*) exposed to dissolved, nano- and bulk-sized silver. Environ Pollut.

[CR11] Dörr AJM, Scalici M, Caldaroni B, Magara G, Scoparo M, Goretti E, Elia AC (2020) Salinity tolerance of the invasive red swamp crayfish Procambarus clarkii (Girard, 1852). Hydrobiologia 847:2065–2081

[CR12] Elia AC, Galarini R, Dörr AJM, Taticchi MI (2007). Heavy metal contamination and antioxidant response of a freshwater bryozoan (*Lophopus crystallinus* Pall., Phylactolaemata). Ecotoxicol Environ Saf.

[CR13] Elia AC, Magara G, Righetti M, Dörr AJM, Scanzio T, Pacini N, Abete MC, Prearo M (2017). Oxidative stress and related biomarkers in cupric and cuprous chloride-treated rainbow trout. Environ Sci Pollut Res.

[CR14] Elia AC, Magara G, Caruso C, Masoero L, Prearo M, Arsieni P, Caldaroni B, Dörr AJM, Scoparo M, Salvati S, Brizio P, Squadrone S, Abete MC (2018). A comparative study on subacute toxicity of arsenic trioxide and dimethylarsinic acid on antioxidant status in Crandell Rees feline kidney (CRFK), human hepatocellular carcinoma (PLC/PRF/5), and epithelioma papulosum cyprini (EPC) cell lines. J Toxicol Environ Health A.

[CR15] Elia AC, Prearo M, Dörr AJM, Pacini N, Magara G, Brizio P, Gasco L, Abete MC (2019). Effects of astaxanthin and canthaxanthin on oxidative stress biomarkers in rainbow trout. J Toxicol Environ Health A.

[CR16] Elia AC, Burioli E, Magara G, Pastorino P, Caldaroni B, Menconi V, Dörr AJM, Colombero G, Abete MC, Prearo M (2020). Oxidative stress ecology on Pacific oyster Crassostrea gigas from lagoon and offshore Italian sites. Sci Total Environ.

[CR17] Filipkowska A, Lubecki L (2016). Endocrine disruptors in blue mussels and sediments from the Gulf of Gdańsk (Southern Baltic). Environ Sci Pollut Res.

[CR18] Gao JM, Wu L, Chen YP, Zhou B, Guo JS, Zhang K, Ouyang WJ (2017). Spatiotemporal distribution and risk assessment of organotins in the surface water of the Three Gorges Reservoir Region, China. Chemosphere.

[CR19] Gao J, Fu P, Chen X, Guo J, Hou X, Zeng J, Chen Z (2020). Fate simulation and risk assessment of TBT and TPhT considering water level fluctuations in the TGR before and after AFS Convention implementation in China. Environ Sci Eur.

[CR20] Goldberg ED (1986). TBT An environmental dilemma. Environment.

[CR21] Harino H, Fukushima M, Kawai S (2000). Accumulation of butyltin and phenyltin compounds in various fish species. Arch Environ Contam Toxicol.

[CR22] Horiguchi T, Kojima M, Kaya M, Matsuo T, Shiraishi H, Morita M, Adachi Y (2002). Tributyltin and triphenyltin induce spermatogenesis in ovary of female abalone, *Haliotis gigantea*. Mar Environ Res.

[CR23] Ishihara Y, Kawami T, Ishida A, Yamazaki T (2012). Tributyltin induces oxidative stress and neuronal injury by inhibiting glutathione S-transferase in rat organotypic hippocampal slice cultures. Neurochem Int.

[CR24] Jahroudi N, Foster R, Price-Haughey J, Beitel G, Gedamu L (1990). Cell-type specific and differential regulation of the human metallothionein genes. Correlation with DNA methylation and chromatin structure. J Biol Chem.

[CR25] Lagadic L, Coutellec MA, Caquet T (2007). Endocrine disruption in aquatic pulmonate molluscs: few evidences, many challenges. Ecotoxicology.

[CR26] Lopes-dos-Santos RMA, Galante-Oliveira S, Lopes E, Almeida C, Barroso C (2014). Assessment of imposex and butyltin concentrations in *Gemophos viverratus* (Kiener, 1834), from São Vicente, Republic of Cabo Verde (Africa). Environ Sci Pollut Res.

[CR27] Lowry OH, Rosebrough NJ, Farr AL, Randall RJ (1951). Protein measurement with the Folin phenol reagent. J Biol Chem.

[CR28] Lyssimachou A, Santos JG, André A, Soares J, Lima D, Guimarães L, Almeida CM, Teixeira C, Castro LF, Santos MM (2017). The mammalian “obesogen”; tributyltin targets hepatic triglyceride accumulation and the transcriptional regulation of lipid metabolism in the liver and brain of zebrafish. PLoS One.

[CR29] Magara G, Elia AC, Syberg K, Khan FR (2018). Single contaminant and combined exposures of polyethylene microplastics and fluoranthene: accumulation and oxidative stress response in the blue mussel, *Mytilus edulis*. J Toxicol Environ Health A.

[CR30] Magara G, Khan FR, Pinti M, Syberg K, Inzirillo A, Elia AC (2019). Effects of combined exposures of fluoranthene and polyethylene or polyhydroxybutyrate microplastics on oxidative stress biomarkers in the blue mussel (*Mytilus edulis*). J Toxicol Environ Health A.

[CR31] Martin RC, Dixon DG, Maguire RJ, Hodson PV, Tkacz RJ (1989). Acute toxicity, uptake, depuration and tissue distribution of tri-n-butyltin in rainbow trout, *Salmo gairdneri*. Aquat Toxicol.

[CR32] Martínez ML, Piol MN, Sbarbati Nudelman N, Verrengia Guerrero NR (2017). Tributyltin bioaccumulation and toxic effects in freshwater gastropods Pomacea canaliculata after a chronic exposure: field and laboratory studies. Ecotoxicology.

[CR33] McGinnis CL, Crivello JF (2011). Elucidating the mechanism of action of tributyltin (TBT) in zebrafish. Aquat Toxicol.

[CR34] Meyer KA, Sullivan CL, Kennedy P, Schill DJ, Teuscher DM, Brimmer AF, King DT (2016). Predation by american white pelicans and double-crested cormorants on catchable-sized hatchery rainbow trout in select Idaho lentic waters. N Am J Fish Manag.

[CR35] Mitra S, Srivastava A, Khandelwal S (2013). Tributyltin chloride induced testicular toxicity by JNK and p38 activation, redox imbalance and cell death in sertoli-germ cell co-culture. Toxicology.

[CR36] Mitra S, Gera R, Singh V, Khandelwal S (2014). Comparative toxicity of low dose tributyltin chloride on serum, liver, lung and kidney following subchronic exposure. Food Chem Toxicol.

[CR37] Nakatsu Y, Kotake Y, Ohta S (2007). Concentration dependence of the mechanisms of tributyltin-induced apoptosis. Toxicol Sci.

[CR38] Nakayama K, Oshima Y, Nagafuchi K, Hano T, Shimasaki Y, Honjo T (2005). Early-life-stage toxicity in offspring from exposed parent medaka, Oryzias latipes, to mixtures of tributyltin and polychlorinated biphenyls. Environ Toxicol Chem.

[CR39] Okoro HK, Fatoki OS, Adekola FA, Ximba BJ, Snyman RG (2016). Spatio-temporal variation of organotin compounds in seawater and sediments from Cape Town harbour, South Africa using gas chromatography with flame photometric detector (GC-FPD). Arab J Chem.

[CR40] Oshima Y, Nirmala K, Yokota Y, Go J, Shimasaki Y, Nakao M, Lee RF, Imada N, Honjo T, Kobayashi K (1998). Accumulation of tributyltin (TBT) in the blood of flounder and dab intraperitoneally administered with TBT. Mar Environ Res.

[CR41] Pacini N, Prearo M, Abete MC, Brizio P, Dörr AJM, Reimschuessel R, Andersen W, Gasco L, Righetti M, Elia AC (2013). Antioxidant responses and renal crystal formation in rainbow trout treated with melamine administered individually or in combination with cyanuric acid. J Toxicol Environ Health A.

[CR42] Padrós J, Pelletier É, Ribeiro CO (2003). Metabolic interactions between low doses of benzo[a]pyrene and tributyltin in arctic charr (*Salvelinus alpinus*): a long-term in vivo study. Toxicol Appl Pharmacol.

[CR43] Quintas PY, Alvarez MB, Arias AH, Garrido M, Marcovecchio JE (2019). Spatiotemporal distribution of organotin compounds in the coastal water of the Bahia Blanca estuary (Argentina). Environ Sci Pollut Res Int.

[CR44] Revathi P, Iyapparaj P, Vasanthi LA, Munuswamy N, Krishnan M (2013). Impact of TBT on the vitellogenesis and sex hormones in freshwater prawn *Macrobrachium rosenbergii* (De Man, 1879). Aquat Biosyst.

[CR45] Revathi P, Iyapparaj P, Vasanthi L, Munuswamy N, Arun Prasanna V, Suganya T, Anantharaman P, Krishnan M (2014). TBT effects on the development of intersex (Ovotestis) in female freshwater prawn *Macrobrachium rosenbergii*. Biomed Res Int.

[CR46] Revathi P, Iyapparaj P, ArockiaVasanthi L, Munuswamy N, Arun V, Pandiarajan J, Krishnan M (2014). Influence of short term exposure of TBT on the male reproductive activity in freshwater prawn *Macrobrachium rosenbergii* (De Man). Bull Environ Contam Toxicol.

[CR47] Ritsema R, Laane RWPM, Donard OFX (1991). Butyltins in marine waters of The Netherlands in 1988 and 1989; Concentrations and effects. Mar Environ Res.

[CR48] Santos DM, Araújo IP, Machado EC, Carvalho-Filho MAS, Fernandez MA, Marchi MRR, Godoi AFL (2009). Organotin compounds in the Paranaguá Estuarine Complex, Paraná, Brazil: evaluation of biological effects, surface sediment, and suspended particulate matter. Mar Pollut Bull.

[CR49] Shimasaki Y, Oshima Y, Inoue S, Inoue Y, Kang IJ, Nakayama K, Imoto H, Honjo T (2006). Effect of tributyltin on reproduction in Japanese whiting, *Sillago japonica*. Mar Environ Res.

[CR50] Teather K, Parrott J (2006). Assessing the chemical sensitivity of freshwater fish commonly used in toxicological studies. Water Qual Res J Can.

[CR51] Tolosa I, Readman JW, Blaevoet A, Ghilini S, Bartocci J, Horvat M (1996). Contamination of Mediterranean (Cote d’Azur) coastal waters by organotins and Irgarol 1051 used in antifouling paints. Mar Pollut Bull.

[CR52] Valkirs AO, Seligman PF, Stang PM, Homer V, Lieberman SH, Vafa G, Dooley CA (1986). Measurement of butyltin compounds in San Diego Bay. Mar Pollut Bull.

[CR53] Wang CG, Chen YX, Li Y, Wei W, Yu Q (2005). Effects of low dose tributyltin on activities of hepatic antioxidant and phase II enzymes in *Sebastiscus marmoratus*. Bull Environ Contam Toxicol.

[CR54] Wang C, Zhao Y, Zheng R, Ding X, Wei W, Zuo Z, Chen Y (2006). Effects of tributyltin, benzo[a]pyrene, and their mixture on antioxidant defense systems in *Sebastiscus marmoratus*. Ecotoxicol Environ Saf.

[CR55] Wu Y, Wang C, Wang Y, Zhao Y, Chen Y, Zuo Z (2007). Antioxidant responses to benzo[a]pyrene, tributyltin and their mixture in the spleen of *Sebasticus marmoratus*. J Environ Sci.

[CR56] Zhang JL, Zuo ZH, He CY, Cai JL, Wang YQ, Chen YX, Wang CG (2009). Effect of tributyltin on testicular development in *Sebastiscus marmoratus* and the mechanism involved. Environ Toxicol Chem.

[CR57] Zhang C, Zhang J, Ren H, Zho B, Wu Q, Sun P (2017). Effect of tributyltin on antioxidant ability and immune responses of zebrafish (*Danio rerio*). Ecotoxicol Environ Saf.

[CR58] Zhang J, Zhang C, Ma D, Liu M, Huang S (2017). Lipid accumulation, oxidative stress and immune-related molecules affected by tributyltin exposure in muscle tissues of rare minnow (*Gobiocypris rarus*). Fish Shellfish Immunol.

[CR59] Zuo Z, Wang C, Wu M, Wang Y, Chen Y (2012). Exposure to tributyltin and triphenyltin induces DNA damage and alters nucleotide excision repair gene transcription in *Sebastiscus marmoratus* liver. Aquat Toxicol.

